# The Impact of Intraoperative Dexamethasone and Mannitol on Postoperative Inflammatory Response in Totally Tubeless Percutaneous Nephrolithotomy: A Prospective, Double‐Blind, Controlled Clinical Trial

**DOI:** 10.1155/aiu/9465166

**Published:** 2026-07-22

**Authors:** Ehsan Zemanati Yar, Iman Menbari Oskouie, Abdolreza Mohammadi, Mahdi Najarzadegan, Mohammadreza Nikoobakht, Roghayeh Koohi Ortakand, Seyed Reza Hosseini, Seyed Mohammad Kazem Aghamir

**Affiliations:** ^1^ Urology Research Center, Tehran University of Medical Sciences, Tehran, Iran, tums.ac.ir; ^2^ Department of Urology, Sina Hospital, Tehran University of Medical Sciences, Tehran, Iran, tums.ac.ir; ^3^ Hematology, Oncology and Stem Cell Transplantation Research Center, Tehran University of Medical Sciences, Tehran, Iran, tums.ac.ir

**Keywords:** dexamethasone, interleukin-6, mannitol, pain management, SIRS, totally tubeless percutaneous nephrolithotomy

## Abstract

**Background and Aims:**

Dexamethasone, a corticosteroid, reduces inflammation, while mannitol, a diuretic, improves renal blood flow and stone clearance. This study examines the impact of dexamethasone and mannitol during surgery on infection rates, pain control, and analgesic needs in totally tubeless PCNL (TTPCNL) patients.

**Methods:**

This double‐blind, controlled clinical trial at a single center involved 121 patients undergoing TTPCNL. Participants were randomly divided into four groups: control, dexamethasone, mannitol, and dexamethasone–mannitol (DM). Postoperative outcomes included pain intensity measured using the visual analog scale (VAS) at 6 and 24 hours after tubleless PCNL. Patients with moderate‐to‐severe pain received rescue analgesia (paracetamol and ketorolac). Additional outcomes included length of hospital stay (LOS), serum interleukin‐6 (IL‐6) levels, gross hematuria, stone‐free rate (SFR), Clavien–Dindo grade (CDG), and postoperative complications, including sepsis, systemic inflammatory response syndrome (SIRS), hemoglobin decline, the need for angiography, hospital readmission, and acute kidney injury (AKI).

**Results:**

This study included 81 male participants (66.9%), with a mean age of 50.9 ± 12.2 years. Patients treated with dexamethasone, either alone or with mannitol, showed significant reductions in postoperative SIRS (*p* value < 0.001), hemoglobin drop (*p* value < 0.001), hospital stay duration (*p* value < 0.001), CDG (*p* value < 0.001), postoperative pain (*p* value < 0.001), and analgesic use (*p* value = 0.015). IL‐6 levels in DM group were significantly lower compared to others (*p* = 0.002). There were no significant reductions in occurrence of AKI, readmission, angiography, sepsis, and SFR.

**Conclusions:**

The intraoperative use of dexamethasone and mannitol during TTPCNL effectively mitigated postoperative inflammatory responses. This was achieved by reducing levels of IL‐6 and decreasing the occurrence of SIRS. Additionally, patients experienced a shorter postoperative hospital stay, lower VAS pain score, lower CDG, and lower analgesic consumption.

**Trial Registration:** Iranian Registry of Clinical Trials (IRCT): IRCT20190305042939N1

## 1. Introduction

The global incidence and prevalence of renal stones have shown a marked increase over recent decades, with an estimated prevalence of approximately 10% in United States alone [1]. In the last 20 years, traditional open surgery for renal stone treatment has been largely replaced by less invasive approaches [2]. According to the guidelines of the European Association of Urology, percutaneous nephrolithotomy (PCNL) is the preferred first‐line treatment for large renal stones (> 2 cm) and multiple stones [3].

The use and removal of nephrostomy tubes in PCNL can lead to complications like infections, pain, urine leaks, bleeding, and longer hospital stays [4]. To address these issues, tubeless PCNL was developed, involving the placement of a double J ureteric stent after the procedure. However, this stent can cause issues such as frequent urination, urgency, nocturia, pain, and blood in the urine [5]. To reduce these stent‐related problems, the concept of totally tubeless PCNL (TTPCNL) was brought back into practice [6].

One of the most frequent complications following PCNL is postoperative infection, with an incidence ranging from 21% to 31.2% [7]. Such infections can lead to urosepsis, a potentially fatal condition, occurring at a rate of 0.3%–4.7% and manifesting as systemic inflammatory response syndrome (SIRS) or progressing to uroseptic shock [8–10]. Urosepsis is a severe condition with a reported mortality rate of 30%–40% [11]. However, despite negative preoperative urine cultures and the use of prophylactic antibiotics, urosepsis remains challenging to prevent and can lead to serious clinical complications [12, 13].

Another significant clinical challenge is postoperative pain following PCNL, primarily due to the distention of the renal capsule and parenchyma [14, 15]. If not properly managed, postoperative pain can result in prolonged recovery periods, extended hospital stays, higher healthcare costs, psychological stress, increased likelihood of chronic postoperative pain, and overall poorer clinical outcomes [16–18]. Therefore, optimizing pain management strategies remains a critical aspect of perioperative care following PCNL [17].

The surgical stress response can amplify the production of inflammatory components. Dexamethasone, a potent corticosteroid, exerts strong anti‐inflammatory effects by inhibiting prostaglandin production and the aggregation of inflammatory complexes [19, 20]. It also reduces the release of lysosomal enzymes and suppresses the synthesis of various inflammatory mediators. With a biological half‐life ranging from 36 to 54 h, dexamethasone plays a critical role in the immediate postoperative period [21]. Additionally, earlier research has shown that dexamethasone is effective and safe for managing postoperative pain, particularly in arthroplasty procedures [22, 23].

Mannitol, a metabolically inert sugar alcohol, shares structural similarities with dextrose. As an osmotic diuretic, it functions primarily by inducing renal vasodilation, enhancing tubular flow, and aiding in the prevention of intratubular cast formation. Additionally, mannitol helps reduce postischemic cellular swelling and may act as a free radical scavenger, offering protective benefits. This effect can be particularly beneficial in promoting the clearance of residual stone fragments or debris from the urinary tract after surgical procedures like PCNL [24, 25]. It has been hypothesized that, in low concentrations, mannitol might exhibit properties similar to dextrose in the context of chronic pain management [26]. Furthermore, intravenous mannitol, when combined with topical steroid treatments or topical gabapentin, has demonstrated positive outcomes in managing complex regional pain syndrome [27, 28].

Currently, there are no definitive guidelines regarding the best practices for managing postoperative SIRS and pain following TTPCNL. Therefore, providing appropriate and effective treatment for both infection and pain after the procedure is crucial for reducing complications, shortening hospital stays, and minimizing healthcare costs. In this clinical trial, we aim to assess the impact of intraoperative administration of dexamethasone and mannitol on postoperative infection rates, pain management, and analgesic use in patients undergoing TTPCNL.

## 2. Materials and Methods

### 2.1. Study Design

This is a prospective, double‐blind, controlled clinical trial conducted at a single center. The trial protocol was reviewed and approved by the Ethics Committee of Tehran University of Medical Sciences (IR.TUMS.SINAHOSPITAL.REC.1403.101). Patients were included after providing written informed consent and registration in the Persian Registry for Stones of the Urinary System (PERSUS) (IRCT20190624043991N32). Prior to participation, all eligible patients or their authorized representatives provided written informed consent.

### 2.2. Participants

The study included patients who underwent TTPCNL at Sina Hospital (Tehran, Iran) between September 2023 and May 2024. The sample size was based on a previously published study by Ramaraju et al. [29], which included a comparable cohort of 120 patients. These patients were randomly assigned to one of four groups: (1) control, (2) dexamethasone, (3) mannitol, and (4) a combination of dexamethasone and mannitol (DM). For those assigned to the dexamethasone group, 8 mg of dexamethasone was added to a normal saline solution and administered at the beginning of anesthesia, following intubation [30]. In the mannitol group, 12.5 g of mannitol was infused intravenously over 20 min [31]. The DM group received both dexamethasone and mannitol as mentioned. TTPCNL was defined as a PCNL procedure performed without the placement of a nephrostomy tube or a ureteral stent at the conclusion of surgery.

The inclusion criteria for the study were as follows: (1) patients scheduled for elective TTPCNL under general anesthesia; (2) patients more than 18 years of age; and (3) the provision of written informed consent. Exclusion criteria included the following: (1) history of surgery for ipsilateral kidney stones; (2) severe urinary tract infections; (3) significant cardiac and pulmonary insufficiency or coagulation disorders; (4) spinal deformities, underlying pathologies, or history of spinal cord injury surgery; (5) neurological or psychiatric disorders; (6) use of corticosteroids or chemotherapy; (7) bilateral kidney stones; (8) the presence of both kidney and ureteral stones simultaneously; (9) any urogenital anomalies; (10) chronic kidney disease (CKD) patients; and (11) any hypersensitivities or contraindications to the administration of mannitol or dexamethasone.

### 2.3. Randomization, Allocation Concealment, and Blinding

Randomization was performed using a computer‐generated random sequence without block restriction. Allocation concealment was achieved using sequentially numbered, sealed, opaque envelopes prepared by an independent investigator not involved in patient recruitment or outcome assessment. This study was conducted in a double‐blind manner, with both patients and outcome assessors blinded to group allocation. The anesthesiologist administering the study medications and the surgical team remained blinded to treatment allocation throughout the procedure.

### 2.4. Interventions

According to standard preoperative fasting protocols, patients were required to abstain from food and liquids for at least 8 h prior to the procedure. A prophylactic dose of the antibiotic ertapenem (1000 mg) was administered 30 min before surgery. Intravenous access was established in the upper extremities, and routine monitoring of blood pressure (BP), electrocardiogram (ECG), heart rate (HR), and oxygen saturation (SpO2) was conducted throughout the procedure.

### 2.5. Outcomes

Participants’ pain levels were evaluated using the visual analog scale (VAS), a widely accepted and validated 10‐point scale for self‐reported pain. On this scale, a score of 0 indicates no pain, while a score of 10 reflects the most severe pain imaginable. VAS assessments were conducted by a well‐trained physician at two key time points: 6 h and 24 h after surgery, while the patients were still in the ward.

### 2.6. Routine Analgesia Protocol and Rescue Analgesia

Participants were categorized into two groups based on their VAS pain scores: those experiencing mild or no pain (VAS score: 0–3) and those with moderate‐to‐severe pain (VAS score: 4–10). Rescue analgesia was administered intravenously following a predefined protocol, with paracetamol (1 g IV) and ketorolac (30 mg IV) given to patients reporting moderate‐to‐severe pain. Both medications were to be administered with a minimum interval of 4 hours between injections.

### 2.7. Data Collection

Upon hospital admission, all participants underwent a comprehensive preoperative evaluation, including their medical history, physical examination, routine laboratory tests, and radiological assessments, such as an abdominal CT scan.

Postoperative assessments (24 h after surgery) were conducted by urologists, covering several key parameters: body temperature (fever), complete blood count (CBC), blood urea nitrogen (BUN), creatinine (Cr) levels, erythrocyte sedimentation rate (ESR), C‐reactive protein (CRP), and interleukin‐6 (IL‐6). Additionally, the duration of hospital stay (LOS), presence of gross hematuria, stone‐free rate (SFR), and postoperative complications such as sepsis, SIRS, hemoglobin drop, need for angiography, readmission, acute kidney injury (AKI), and ICU admission were documented. The Clavien–Dindo system is used for grading adverse events that occur as a result of TTPCNL [32].

The objective diagnosis of SIRS was based on the presence of any two of the following criteria [33]:

• Body temperature higher than 38°C or lower than 36°C.
• HR exceeding 90 beats per minute.
• Respiratory rate greater than 20 breaths per minute or a partial pressure of CO_2_ below 32 mmHg.
• A leukocyte count greater than 12,000 or lower than 4000 cells/μL or the presence of more than 10% immature forms (bands).

### 2.8. Statistical Analysis

For the statistical analysis, IBM SPSS Statistics Version 25.0 (IBM Corp., Armonk, NY, USA) was utilized. The Kolmogorov–Smirnov test was employed to assess whether continuous variables adhered to a normal distribution before proceeding with further comparisons. Data that followed a normal distribution were expressed as mean ± standard deviation (SD), whereas nonnormally distributed data were summarized as median ± interquartile range (IQR). The ANOVA test was used to compare normally distributed groups, while the Kruskal–Wallis test was applied to nonnormally distributed groups. For pairwise comparisons within normal groups post‐ANOVA, the Bonferroni correction was applied, and for nonnormal groups post‐Kruskal–Wallis test, the Mann–Whitney *U* test was used. Qualitative variables were compared using the chi‐square test. A *p* value of less than 0.05 was considered statistically significant for detecting differences between groups.

## 3. Results

This study included a total of 121 patients, of which 81 (66.9%) were male. The mean age of the participants was 50.9 ± 12.2 years, and their body mass index (BMI) was 27.6 ± 2.6 kg/m^2^. There were no statistically significant differences in baseline characteristics such as gender, age, BMI, kidney side, or stone location across the different treatment groups, with all *p* values exceeding 0.05. Furthermore, the results of the physical examinations and the basic laboratory tests were comparable among the groups, showing no significant difference. Detailed information on the characteristics of the patients is provided in Table 1.

**TABLE 1:** Patient characteristics in different groups of intervention, mean (SD) or frequencies (%).

**Variable**		**Total (*n* = 121)**	**Control (*n* = 30)**	**Dexamethasone (*n* = 33)**	**Mannitol (*n* = 28)**	**DM (*n* = 30)**	**p** **value**

Sex	Female	40 (33.1%)	10 (33.3%)	9 (27.3%)	9 (32.1%)	12 (40.0%)	0.762
	Male	81 (66.9%)	20 (66.7%)	24 (72.7%)	19 (67.9%)	18 (60.0%)	

Age	Years	50.9 (12.2)	54.9 (12.0)	47.5 (10.2)	52.9 (13.1)	48.9 (12.7)	0.058

BMI	kg/m^2^	27.6 (2.6)	27.6 (2.2)	28.5 (2.4)	28.2 (2.3)	26.9 (2.8)	0.061

Side	Left	69 (57.0%)	22 (73.3%)	18 (54.5%)	12 (42.9%)	17 (56.7%)	0.131
	Right	52 (43.0%)	8 (26.7%)	15 (45.5%)	16 (57.1%)	13 (43.3%)	

Stone location	Pelvis	95 (78.5%)	23 (76.7%)	24 (72.7%)	25 (89.3%)	23 (76.7%)	
	Middle calyx	5 (4.1%)	1 (3.3%)	1 (3.03%)	2 (7.1%)	1 (3.3%)	0.941
	Inferior calyx	59 (48.8%)	9 (30%)	18 (54.5%)	15 (53.6%)	17 (56.7%)	
	Superior calyx	17 (14.0%)	6 (20%)	5 (15.1%)	4 (14.3%)	2 (6.7%)	

Abbreviations: BMI, body mass index; DM, dexamethasone and mannitol.

Table 2 presents the postoperative physical examination findings and laboratory results across the various study groups. Notably, the incidence of fever was significantly lower in the dexamethasone and DM groups compared to the other groups (*p* < 0.001). Additionally, patients who received dexamethasone, either alone or in combination with mannitol, exhibited significantly reduced levels of WBC, BUN, Cr, ESR, and CRP, with all *p* values < 0.001.

**TABLE 2:** Comparison of postoperative (24 h) physical examination and laboratory data in different groups.

**Variable**		**Total (*n* = 121)**	**Control (*n* = 30)**	**Dexamethasone (*n* = 33)**	**Mannitol (*n* = 28)**	**DM (*n* = 30)**	**p** **value**

SBP	mmHg	127.5 (11.5)	131.3 (15.2)	123.9 (5.5)	131.5 (13.2)	124.0 (7.7)	0.005^∗^

DBP	mmHg	76.7 (6.3)	79.3 (6.4)	76.1 (5.0)	76.0 (7.0)	75.3 (6.3)	0.06

HR	Pulse/min	82.2 (7.4)	87.4 (7.53)	78.4 (4.5)	85.3 (8.0)	78.3 (4.6)	< 0.001^∗^

Fever	No	77	6	31	10	30	< 0.001^∗^
	Yes	44	24	2	18	0	

WBC	U/μL	11.34 (3.4)	13.5 (3.3)	9.3 (2.0)	12.7 (3.9)	10.2 (2.23)	< 0.001^∗^

HB	g/dL	12.4 (1.6)	12.0 (1.7)	13.1 (1.2)	11.6 (1.8)	12.9 (1.1)	< 0.001^∗^
PLT	U/μL	198.0 (67.4)	201.4 (61.2)	211.5 (73.2)	195.9 (63.9)	182.0 (69.5)	0.376

BUN	mg/dL	33.6 (28.5)	38.2 (16.9)	27.3 (6.9)	32.1 (12.7)	24.7 (5.9)	< 0.001^∗^

Cr	mg/dL	1.12 (0.48)	1.42 (0.66)	1.02 (0.23)	1.17 (0.53)	0.86 (0.19)	< 0.001^∗^

ESR	mm/h	25.1 (17.8)	31.6 (16.9)	20.3 (13.8)	33.8 (23.1)	15.9 (9.2)	< 0.001^∗^

CRP	mg/L	53.6 (28.4)	72.3 (24.7)	42.4 (24.8)	60.5 (30.6)	40.7 (21.4)	< 0.001^∗^

*Note:* Mean (SD) or frequencies (%). WBC: white blood cell count; HB: hemoglobin; PLT: platelets; Cr: creatinine.

Abbreviations: BUN, blood urea nitrogen; CRP, C‐reactive protein; DBP, diastolic blood pressure; DM, dexamethasone and mannitol; ESR, erythrocyte sedimentation rate; HR, heart rate; SBP, systolic blood pressure.

^∗^Significant difference.

The study found that the occurrence of postoperative gross hematuria, SIRS, and hemoglobin drop was significantly reduced in the dexamethasone and DM groups (*p* < 0.001). These patients also experienced a significantly shorter hospital stay (*p* < 0.001). Postoperative pain levels at both 6 and 24 h were significantly lower in patients who received dexamethasone, with or without mannitol, compared to other groups (*p* < 0.001). Furthermore, the consumption of analgesics (*p* = 0.015) and the Clavien–Dindo grade of complications were significantly reduced in these groups (*p* < 0.001). IL‐6 levels were markedly lower in the DM group than in other groups (*p* = 0.002).

Although the chance of requiring postoperative angiography, readmission, AKI, sepsis, and ICU admission was reduced in these groups, these differences were not statistically significant. The SFR did not significantly differ among the various groups (Table 3). It is noteworthy that for these outcomes with very low event rates (sepsis, angiography, readmission, AKI, and ICU admission), statistical comparisons were not feasible due to small sample sizes; therefore, these were reported as “NV” (no valid *p* value).

**TABLE 3:** Comparison of outcomes and complications in different groups: mean (SD), median (IQR), or frequencies (%).

**Variable**		**Total (*n* = 121)**	**Control (*n* = 30)**	**Dexamethasone (*n* = 33)**	**Mannitol (*n* = 28)**	**DM (*n* = 30)**	**p** **value**

Gross hematuria	No	89 (73.5%)	18 (60%)	30 (90.9%)	12 (42.9%)	29 (96.7%)	< 0.001^∗^
	Yes	32 (26.5%)	12 (40%)	3 (9.1%)	16 (57.1%)	1 (3.3%)	

SFR	No	27	12	5	5	5	0.064
	Yes	94	18	28	23	25	

Hospital stay	Days	4.17 (2.1)	5.83 (2.35)	3.12 (0.99)	5.25 (2.01)	2.67 (0.48)	< 0.001^∗^

SIRS	No	78 (64.5%)	6 (20%)	32 (97.0%)	10 (35.7%)	30 (100%)	< 0.001^∗^
	Yes	43 (35.5%)	24 (80%)	1 (3.0%)	18 (64.3%)	0 (0%)	

Hemoglobin drop	No	99 (81.8%)	19 (63.3%)	31 (93.9%)	19 (67.9%)	30 (100%)	< 0.001^∗^
	Yes	22 (18.2%)	11 (36.7%)	2 (6.1%)	9 (32.1%)	0 (0%)	

Sepsis	No	113 (93.4%)	25 (83.3%)	31 (93.9%)	27 (96.4%)	30 (100%)	NV
	Yes	8 (6.6%)	5 (16.7%)	2 (6.1%)	1 (3.6%)	0 (0%)	

Angiography	No	117 (96.7%)	27 (90%)	33 (100%)	27 (96.4%)	30 (100%)	NV
	Yes	4 (3.3%)	3 (10%)	0 (0%)	1 (3.6%)	0 (0%)	

Readmission	No	119 (98.3%)	28 (93.3%)	33 (100%)	28 (100%)	30 (100%)	NV
	Yes	2 (1.7%)	2 (6.7%)	0 (0%)	0 (0%)	0 (0%)	

AKI	No	116 (95.9%)	27 (90%)	33 (100%)	26 (92.9%)	30 (100%)	NV
	Yes	5 (4.1%)	3 (10%)	0 (0%)	2 (7.1%)	0 (0%)	

Number of analgesic transfusions	n	1 (1–3)	3 (3–3)	1 (1–1)	2.5 (2–3)	1 (1–1)	0.015^∗^

Pain score	6 h	6.21 (2.00)	7.93 (0.83)	4.58 (1.41)	7.89 (0.92)	4.70 (1.32)	< 0.001^∗^

Pain score	24 h	2.07 (1.87)	4.03 (0.85)	0.58 (0.75)	3.61 (0.83)	0.33 (0.71)	< 0.001^∗^

ICU admission	No	117	28	33	26	30	NV
	Yes	4	2	0	2	0	

IL‐6	pg/mL	29.4 (5.5)	33.3 (5.9)	29.1 (3.9)	32.1 (4.2)	25.3 (3.2)	0.002^∗^

Clavien–Dindo grade	I	92 (76.0%)	17 (56.7%)	31 (93.9%)	14 (50.0%)	30 (100.0%)	< 0.001^∗^
	II	20 (16.5%)	8 (26.7%)	2 (6.1%)	10 (35.7%)	0	
	III	7 (5.8%)	4 (13.3%)	0 (0%)	3 (10.7%)	0	
	IV	2 (1.6%)	1 (3.3%)	0 (0%)	1 (3.6%)	0	

*Note:* IL‐6, interleukin‐6. NV: no valid *p* value due to small sample size.

Abbreviations: AKI, acute kidney injury; DM, dexamethasone and mannitol; ICU, intensive care unit; SIRS: systemic inflammatory response syndrome.

^∗^Significant difference.

## 4. Discussion

The global increase in renal calculi across all age groups is largely due to changes in dietary habits and the effects of global warming [34]. Currently, PCNL is considered the preferred treatment for large or multiple kidney stones, especially staghorn calculi, and is becoming increasingly accepted by urologists. However, postoperative fever, with an incidence rate of 21%–32.1%, remains a major concern, as it can develop into life‐threatening urosepsis [35, 36]. In patients with urolithiasis, bacteria are commonly found not only in the urine but also within the stones themselves, which include both infectious and metabolic types. During PCNL, the fragmentation of stones can release these bacteria and endotoxins. Additionally, the hydraulic pressure from the irrigation fluid used during the procedure can push these bacteria and endotoxins into the circulatory system, increasing the risk of postoperative infections [37]. Postoperative pain is another significant challenge, with around 60% of patients experiencing moderate to severe pain after PCNL, according to Wu et al. [38].

Dexamethasone, a long‐acting glucocorticoid, has anti‐inflammatory, immunosuppressive, and antipyretic properties [39], while mannitol, a diuretic, may help reduce the absorption of bacteria and endotoxins, potentially lowering infection risks [24, 25]. Despite their potential benefits, to date, no studies have evaluated the combined use of dexamethasone and mannitol for postoperative anti‐inflammatory effects in PCNL patients.

The current study demonstrated that patients in the dexamethasone and DM groups reported improved VAS pain scores at both 6 and 24 h postoperatively (*p* < 0.001), along with a reduced need for analgesics (*p* = 0.015). Furthermore, there was a significant reduction in the incidence of gross hematuria, SIRS, and hemoglobin drop, as well as a shorter hospital stay duration and lower Clavien–Dindo grading (CDG) (*p* < 0.001). Although the probability of sepsis, angiography, hospital readmission, AKI, and ICU admission also decreased, these reductions were not statistically significant. Given the relatively low incidence of these complications and the limited sample size, the study may have been underpowered to detect significant differences for these outcomes; therefore, these findings should be interpreted with caution and warrant further investigation in larger studies. The observed improvements in inflammatory markers and clinical recovery may be related to the anti‐inflammatory effects of dexamethasone and mannitol, which may help attenuate postoperative inflammatory responses.

Qi et al. have investigated the perioperative use of dexamethasone combined with furosemide to reduce inflammation in patients undergoing PCNL. Their study involved 147 participants and found that the incidence of urosepsis was significantly lower in the treatment group (11.69%) compared to the control group (24.29%, *p* = 0.046). Additionally, patients in the dexamethasone–furosemide group experienced shorter postoperative hospital stays (4.45 ± 1.561 days vs. 5.09 ± 2.083 days, *p* = 0.041) [40]. These findings support our study, where the DM group showed similarly reduced hospitalization times (2.67 ± 0.48 days vs. 5.83 ± 2.35 days, *p* < 0.001), as well as a significantly lower incidence of SIRS (0% vs. 80%, *p* < 0.001).

Inflammation involves a complex balance of proinflammatory and anti‐inflammatory processes, marked by the release of cytokines like IL‐6 [41]. IL‐6 is a multifunctional cytokine produced by cells such as T lymphocytes, fibroblasts, and macrophages during inflammation, tissue necrosis, or tumor growth [42]. It plays a critical role in driving the inflammatory response and is strongly associated with infection severity, making it a reliable marker for predicting serious infections [43]. In our study, patients treated with DM had significantly lower postoperative IL‐6 levels compared to other groups (*p* = 0.002), indicating reduced infection severity and inflammation. Qi et al. also observed similar findings, where patients in the dexamethasone and furosemide group showed lower IL‐6 levels at 2 hours post‐surgery (*p* = 0.045) and on postoperative day one (*p* = 0.031), compared to the control group. This supports the effectiveness of these treatments in controlling inflammation and infection [40].

During PCNL, bacteria and endotoxins within the stone are released when the stone fragments and the pressure from the irrigation fluid can transport them into the circulatory system through the damaged renal mucosa. This process leads to infection and triggers inflammation. Inflammation is initiated at the site of injury through activation of macrophages and mast cells, leading to the release of proinflammatory mediators like cytokines, lipid mediators, and bioactive amines. This activates vasodilation, increases capillary permeability, and causes leukocyte aggregation, which can result in a systemic inflammatory response [44]. Mannitol, a strong diuretic, enhances urine production, limiting the absorption of bacteria and endotoxins, thereby reducing the risk of infection and inflammation. Dexamethasone, known for its anti‐inflammatory and antipyretic effects, suppresses the release of proinflammatory mediators such as cyclooxygenase‐2, IL‐1, IL‐6, and TNF‐γ, ultimately diminishing the inflammatory response. A single dose of dexamethasone can also activate endothelial nitric oxide synthase, helping to mitigate the systemic inflammatory response via a nongenomic signaling pathway [45].

Postoperative pain following PCNL can significantly hinder a patient’s recovery, affecting their daily activities, quality of life, and even socioeconomic factors [46]. Insufficient pain management may lead to complications such as increased morbidity, impaired breathing, and longer hospital stays, which, in turn, drive up medical costs. Additionally, if acute pain is not properly controlled, it can evolve into chronic pain, presenting a long‐term challenge to the patient’s rehabilitation and overall well‐being [47].

Al Demour et al. conducted a study on 50 PCNL patients, randomly assigning them into two groups. The study group (*n* = 25) received 20 mL of 0.25% bupivacaine infiltration along the nephrostomy tract, while the control group (*n* = 25) did not receive any infiltration. Postoperative pain was measured using VAS at various intervals. After 8 hours, the study group showed significantly lower pain scores compared to the control group (5.1 ± 1.4 vs. 6.9 ± 1.4, *p* < 0.001) [48] Similarly, Ucar et al. studied 50 patients scheduled for PCNL, using two different pain management strategies: Group 1 received 800 mg IV ibuprofen and Group 2 received 1 g IV paracetamol. Both groups showed similar results in terms of pain intensity, sedation, nausea, vomiting, itching, need for additional analgesia, and satisfaction. The median (IQR) VAS score at 6 h was 3 (2–4) for Group 1 and 2 (2–4) for Group 2 [49]. These findings align with our study, where the mean VAS score at 6 h was 4.7 ± 1.32 in the DM group and 7.93 ± 0.83 in the control group.

This study has several limitations. First, this study was conducted at a single tertiary referral center, which may limit the generalizability and external validity of the findings to other patient populations and clinical settings. While this design allowed for standardized surgical and perioperative protocols, potentially reducing variability and confounding factors, the results should be extrapolated with caution, and larger multicenter studies are warranted for external validation. Second, the relatively small sample size may have limited the statistical power of the study, particularly for detecting differences in infrequent clinical events such as sepsis and AKI. As a result, the study may have been underpowered for these rare secondary outcomes, and the possibility of Type II errors cannot be excluded. Therefore, findings related to these complications should be interpreted with caution.

Third, the follow‐up period was limited to the early postoperative phase, with inflammatory markers and pain outcomes assessed within 24 h after surgery. Also, the approach used for sample size determination may limit the statistical robustness of the findings. Therefore, the long‐term effects of dexamethasone and mannitol on recovery, complications, and pain control remain unclear. Future multicenter studies with larger sample sizes and extended follow‐up are warranted to validate and expand upon these findings.

## 5. Conclusion

This prospective, double‐blind clinical trial suggests that intraoperative administration of dexamethasone, particularly in combination with mannitol, may attenuate postoperative inflammatory response following TTPCNL, as reflected by reduced IL‐6 levels, lower incidence of SIRS, improved pain control, and shorter hospital stay. However, no significant differences were observed in less frequent complications such as sepsis and AKI, and these outcomes should be interpreted with caution due to their low incidence and limited statistical power. Larger multicenter randomized studies are warranted to confirm these results and further define the clinical role of this intervention.

## Author Contributions

Ehsan Zemanati Yar and Iman Menbari Oskouie: writing–original draft; Abdolreza Mohammadi, Mahdi Najarzadegan, Mohammadreza Nikoobakht, and Roghayeh Koohi Ortakand: validation and methodology; Seyed Reza Hosseini: data analysis; and Seyed Mohammad Kazem Aghamir: conceptualization.

## Funding

There is no funding.

## Ethics Statement

The Ethics Committee of Tehran University of Medical Sciences assessed and authorized the protocol for the current study (IR.TUMS.SINAHOSPITAL.REC.1403.101).

## Conflicts of Interest

The authors declare no conflicts of interest.

## Data Availability

The authors confirm that the data supporting the findings of this study are available upon reasonable request from the corresponding author.
